# Trabectedin for desmoplastic small round cell tumours: a possible treatment option?

**DOI:** 10.1186/2045-3329-4-3

**Published:** 2014-04-25

**Authors:** Anna Maria Frezza, Jeremy S Whelan, Palma Dileo

**Affiliations:** 1 The London Sarcoma Service, University College Hospital, 1st Floor Central, 250 Euston Road, London NW1 2PG, UK; 2 Medical Oncology, University Campus Bio-Medico, Rome, Italy

**Keywords:** Sarcoma, Desmoplastic small round cell tumor, Trabectedin, Chemotherapy

## Abstract

**Background:**

Desmoplastic small round cell tumour (DSRCT) is a rare sarcoma typically affecting young males and usually widely metastatic at presentation. Despite multimodal treatment approaches, the prognosis for DSRCT is extremely poor. Alkylator- and anthracyclines- based regimens are widely used as therapy and an initial response is common. Durable responses are exceptionally rare so further systemic treatment options for these patients represent an unmet medical need. We report two cases of metastatic, pretreated DSRCT patients achieving disease stabilisation with Trabectedin.

**Methods:**

Retrospective review of 2 patients with progressive DSRCT, treated with Trabectedin.

**Results:**

Two males aged 19 and 23 years treated with Trabectedin, 1.5 mg/m^2^ over 24 hours 3 weekly for 6 and 5 cycles respectively. Best responses were stable disease in patient 1 and partial response (RECIST 1.1) in patient 2. Progression free survival was 4 months in both cases. Persistent neutropenia required 4 weekly administration in one patient but no other grade 3-4 toxicities occurred.

**Conclusions:**

This report supports Trabectedin to be active and safe in pre-treated DSRCT patients. Further prospective and collaborative efforts are desirable to better define its role in the management of this disease.

## Background

DSRCT are an extremely rare mesenchymal malignancy, with an estimated annual incidence rate of 0.1 case per 1.000.000. Nine out of ten cases occur in caucasian males aged between 15 and 30 years [1]. DSRCT usually arise and spread in the serosal surfaces (especially within the peritoneal cavity), but can metastasize to the liver, abdominal lymph nodes and lungs. Histologically, they are a member of the family of small round blue cell tumors, characterized by nests of small neoplastic cells surrounded by a prominent desmoplastic stroma [2]. Molecular analysis plays a major role in the diagnosis of DSRCT: 96-97% of all cases harbour a t(11;22) translocation, creating the fusion of EWSR1 gene, on chromosome 22, with the WT1 gene, on chromosome 11 [3].

Due to the extreme rarity of this disease and the lack of prospective studies, there is no consensus regarding the best treatment approaches in DSRCT. DSRCT have been reported to be sensitive to alkylator-based chemotherapy and, despite the absence of a defined standard, aggressive approaches including attempted total macroscopic tumor excision combined with chemotherapy and radiation are commonly used [4-7]. Prognosis remains extremely poor and the identification of further treatment options for DSRCT is an unmet medical need.

## Case presentation

This is the report of the clinical outcome of two patients affected by DSRCT treated with Trabectedin at University College of London Hospital. Data were retrospectively collected and imaging was reviewed.

### Case 1

In May 2010 a 23-year-old male patient, with no significant past medical history, presented with dysuria, low abdominal pain and weight loss. Imaging, including a CT scan of abdomen and pelvis showed a 12 cm large lobulated mass arising from the retro-vescical space with marked peritoneal disease and moderate ascites. Liver and pleural metastases were present. Biopsy of the peritoneal mass was consistent with DSRCT (desmin and pan-cytokeratin positive; cromogranin, CD117, CD56, S100, CK20, CK7, LCA and actin negative). Between June 2010 and February 2011 he received 14 alternating cycles of Vincristine, Cyclophosphamide, Doxorubicin and Ifosfamide, Etoposide (VDC/IE), with a partial radiological response. Exploratory laparoscopy was attempted in March 2011, but no effective debulking was achievable. Between March and July 2011 the patient received maintenance chemotherapy with Vincristine, Actinomycin D and Cyclophosphamide (VAC), discontinued after 6 cycles due to progressive disease. Between August and September 2011 he received treatment with Gemcitabine and Docetaxel, discontinued after two cycles due to rapid clinical progression (worsening of abdominal pain and shortness of breath). With the view to control symptoms, he was promptly started on Trabectedin 1.5 mg/m^2^ given as a 24 hours infusion every 3 weeks (cycle 1 in October 2011). Treatment was tolerated with minimal side effects (grade 2 increase in creatin-phosphokinase). After three cycles the patient reported an improvement in pain control and breathlessness and a radiological stabilisation of disease. Deterioration in symptoms and disease progression was evident after 6 cycles and treatment was discontinued. The patient died in May 2012.

### Case 2

In September 2010 a 19-year-old boy presented with shortness of breath, weight loss, abdominal pain and marked cholestatic jaundice (bilirubin 240 umol/L). A CT scan of abdomen and pelvis showed a large peritoneal mass causing extrinsic compression of rectum, bladder and the portal vein. The tumour caused liver infiltration with hilar obstruction and marked dilatation of intra-hepatic bile ducts. The abdominal mass extended into the thorax through the foramen of Morgagni. A massive right sided pleural effusion, pleural implants and multiple intraparenchymal and serosal liver metastases were reported. The large peritoneal mass was biopsied and pathology was found to be consistent with intrabdominal DSRCT (desmin positive, cytokeratine, NSE, calretinin, S100, LCA and WT1 negative).

**Figure 1:**
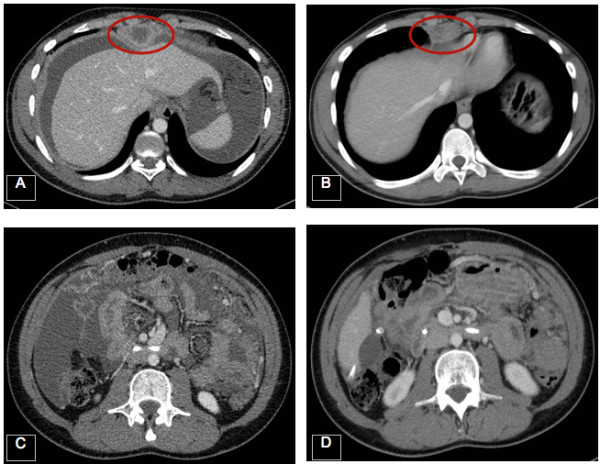
**Activity of Trabectedin in DSRCT.** Reduction in size of a nodule deep to the xiphisternum **(A and B)** and significant reduction of the omental disease **(C and D)** after three cycles of Trabectedin in case 2.

He underwent a therapeutic endoscopic retrograde cholangiopancreatography with stent insertion, pleural effusion drainage and was started on VDC/IE. He completed 14 cycles in March 2011 reporting minimal side effects and achieving a major radiological response and symptomatic relieve with resolution of pain. Between May 2011 and May 2012 he received 12 cycles of VAC before discontinuation at the patient request. In August 2012 he became unwell with marked disease progression and commenced Trabectedin. Treatment was well tolerated with minimal side effects including a grade 1 nausea, grade 2 alkaline phosphatase (437 IU/L) and grade 3 neutropenia (800/mL). A restaging assessment performed in November 2012 after 3 cycles of Trabectedin showed a reduction in size of the nodule deep to the xiphisternum (5 × 2.5 cm) and a significant reduction of the omental disease associated with minimal free fluid in the abdomen and pelvis (Figure 1). Given the radiological response, the patient received two further cycles of Trabectedin, switching to a 4 weekly schedule in view of persistent myelosuppression before disease progression. In November 2012. He failed to benefit from oral cyclophosphamide and prednisolone, dying of disease in January 2013.

## Conclusions

Trabectedin is a marine-derived antineoplastic agent, initially isolated from the tunicate Ecteinascidia turbinata and now produced synthetically, which is currently approved in Europe for the treatment of advanced soft tissue sarcoma after prior treatment with an anthracycline. The first phase II studies were conducted in metastatic leiomyosarcoma and liposarcoma. Myxoid liposarcoma (MLS), a subtype characterised by the expression of the oncogenic transcript FUS/CHOP, has been shown to be extremely sensitive to Trabectedin, with a reported overall response rate of 50% and a median PFS of 17 months [8]. Trabectedin exerts its activity in MLS not only through a direct growth inhibition, but also by affecting the tumour microenvironment and inducing maturation of MLS lipoblasts by targeting the FUS/CHOP-mediated transcriptional block [9,10]. Grohar et al. reported on Trabectedin interfering with the activity of EWS-FLI1 in Ewing sarcoma cells [11]. Activity has been reported in patients with metastatic synovial sarcoma [12], alveolar soft part sarcoma, and endometrial stromal sarcoma. On the basis of Trabectedin activity in translocation related sarcoma, a phase III randomised trial in first-line to compare Trabectedin with doxorubicin-based chemotherapy was initiated and results are awaited.

The potential efficacy of Trabectedin in DSRCT was first postulated by Lopez-Gonzalez A. et al., reporting the case of a patient achieving a partial response with 3-weekly Trabectedin as a third line treatment [13]. More recently, a DSRCT patient treated in the context of a phase I and pharmacokinetic study of Trabectedin in children and adolescents reported stable disease for 6 cycles. The same study showed how Trabectedin, 1.5 mg/m^2^ given as a 24 hour infusion every 3 weeks is safe in adolescents, the toxicity profile being similar to that reported for adults (myelosuppression, elevated serum transaminases, fatigue, nausea and emesis) [14].

In this report we confirmed that Trabectedin can be safely administered in heavily pretreated DSRCT patients, and may provide valuable control of symptoms, albeit temporarily, with radiological stabilization and regression of disease. Tolerability was favorable and toxicities were managed through schedule adjustment without the need for dose reduction. Our results together with data available in literature suggest that Trabectedin is a potentially active treatment option for DSRCT patients and therefore could be considered as a possible further line of treatment at the time of progression after standard regimens.

**Figure 2:**
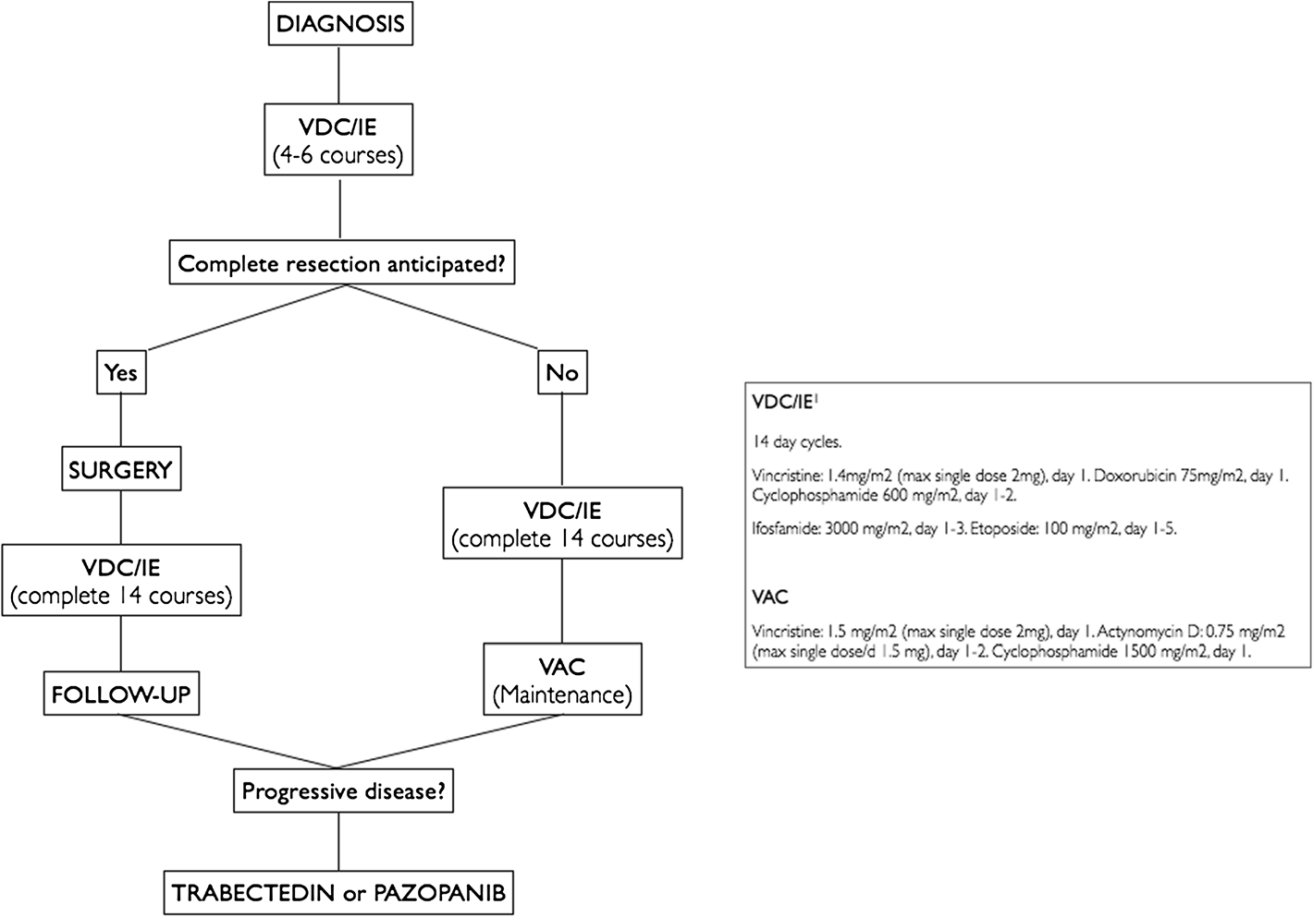
**Institutional standard approach for DSRCT.** Fourteen cycles of interval compressed VDC/IE are administered, with surgery performed at best response (after 4-6 cycles) if complete resection is anticipated. Patients who are not suitable for surgery undergo a maintenance treatment with VAC up to progression or unacceptable toxicity. At time of progression, a single agent treatment with Trabectedin or Pazopanib is considered [15].

To date, no specific evidence-based consensus guidelines have been published for the management of DSRCT. The treatment strategy in our institution is shown in Figure 2 and can provide the basis for prospective evaluation of new approaches and new agents [15]. In view of the rarity of DSRCT and the lack of improvement in outcome, we believe that multi-institutional prospective studies should be promoted to identify better therapeutic approaches including assessment of the value of agents such as Trabectedin.

## Consent

Since both patients died at the time of data review and publication, no written consensus could be obtained.

## Abbreviations

DSRCT: Desmoplastic small round cell tumours; CT: Computed tomography; PET/CT: [F-18]-fluorodeoxy-D-glucose positron emission tomography; VDC/IE: Vincristine, Cyclophosphamide, Doxorubicin and Ifosfamide, Etoposide; VAC: Vincristine, Actinomycin D and Cyclophosphamide.

## Competing interests

AMF received travel coverage for medical meetings from Pharmamar. PD received travel coverage for medical meetings from Pharmamar. JW has no competing interests.

## Authors’ contributions

AMF, PD and JW conceived the paper and participated in collecting data and drafting the manuscript. All the authors read and approved the final manuscript.
